# Local Heterozygosity Effects on Nestling Growth and Condition in the Great Cormorant

**DOI:** 10.1007/s11692-015-9339-2

**Published:** 2015-07-22

**Authors:** Piotr Minias, Katarzyna Wojczulanis-Jakubas, Robert Rutkowski, Krzysztof Kaczmarek

**Affiliations:** Department of Teacher Training and Biodiversity Studies, University of Łódź, Banacha 1/3, 90-237 Łódź, Poland; Department of Biological Sciences, University of Wisconsin-Milwaukee, Milwaukee, WI 53201 USA; Department of Vertebrate Ecology and Zoology, University of Gdańsk, Wita Stwosza 59, 80-308 Gdańsk, Poland; Department of Molecular and Biometrical Techniques, Museum and Institute of Zoology PAS, 00-679 Warsaw, Poland; Medical University of Łódź, Sterlinga 1/3, 91-425 Łódź, Poland

**Keywords:** Great cormorant, Growth rate, Heterozygosity-fitness correlations, Microsatellites, *Phalacrocorax carbo sinensis*

## Abstract

Under inbreeding, heterozygosity at neutral genetic markers is likely to reflect genome-wide heterozygosity and, thus, is expected to correlate with fitness. There is, however, growing evidence that some of heterozygosity-fitness correlations (HFCs) can be explained by ‘local effects’, where noncoding loci are at linkage disequilibrium with functional genes. The aim of this study was to investigate correlations between heterozygosity at seven microsatellite loci and two fitness-related traits, nestling growth rate and nutritional condition, in a recently bottlenecked population of great cormorant *Phalacrocorax carbo sinensis*. We found that heterozygosity was positively associated with both nestling traits at the between-brood level, but the individual (within-brood) effects of heterozygosity were non-significant. We also found that only one locus per trait was primarily responsible for the significant multi-locus HFCs, suggesting a linkage disequilibrium with non-identified functional loci. The results give support for ‘local effect’ hypothesis, confirming that HFCs may not only be interpreted as evidence of inbreeding and that genetic associations between functional and selectively neutral markers could be much more common in natural populations than previously thought.

## Introduction

Genotype-phenotype associations are often complex and difficult to disentangle, but the impact of individual genetic variation on phenotypic quality and fitness has long been recognized as an important evolutionary mechanism. Although heterozygosity has been reported to correlate with fitness-related traits such as reproductive output (Slate et al. 2000; Seddon et al. 2004; Ortego et al. 2009), survival (Markert et al. 2004; Jensen et al. 2007), parasite resistance (MacDougall-Shackleton et al. 2005; Acevedo-Whitehouse et al. 2006), competitive ability (Välimäki et al. 2007; Minias et al. 2015), developmental stability (Vangestel et al. 2011) and quality of ornamentation (Aparicio et al. 2001; Herdegen et al. 2014), the strength of the heterozygosity-fitness correlations (HFCs) is usually weak and it is still difficult to assess the generality of these associations in natural populations (Chapman et al. 2009). It is also acknowledged that HFCs may arise through several different mechanisms, but there is no clear consensus on their relative importance (Hansson and Westerberg 2002).

Heterozygosity at functional loci may affect fitness through overdominance (‘direct effect’ hypothesis), when heterozygous individuals have an intrinsically higher fitness than homozygotes (David 1998). This mechanism cannot, however, easily explain correlations between fitness and heterozygosity at noncoding markers, such as microsatellite loci. Heterozygosity at neutrally selected loci has been assumed to correlate with fitness only in inbred populations, where it is expected to reflect genome-wide heterozygosity, which in turn should correlate with individual inbreeding coefficient (Coulson et al. 1998; Slate et al. 2000). This mechanism is recognized as a ‘general effect’ of heterozygosity and it was initially suggested to explain a large majority of all HFCs reported for noncoding markers. This interpretation was recently challenged by studies showing a weak association between the inbreeding coefficient and heterozygosity measured across a large number of neutrally selected loci (Balloux et al. 2004; Markert et al. 2004; Slate et al. 2004). An association between heterozygosity and inbreeding was suggested to occur only when the variation in individual inbreeding coefficients within populations is high (Slate et al. 2004) or when populations are strongly substructured (Balloux et al. 2004). However, it seems that these specific conditions rarely occur in the wild and, thus, they are unlikely to explain most of the HFCs reported in empirical studies on vertebrates (Pemberton 2004).

As an alternative to the ‘general effect’ hypothesis, it was proposed that heterozygosity at neutral markers can have a ‘local effect’ on fitness, assuming that it is correlated to heterozygosity at both linked and unlinked selected loci through genetic associations (Ohta 1971; Hansson and Westerberg 2002; Szulkin et al. 2010). This hypothesis, however, requires particular population structure and specific evolutionary or ecological circumstances such as small population size, non-random mating, population admixture, or bottlenecks, which can generate non-random associations of alleles at different loci, known as linkage disequilibria (Brouwer et al. 2007; Szulkin et al. 2010). Although linkage disequilibria were initially considered to be restricted to a narrow chromosomal segment around the target locus, it is now realized that high levels of genome-wide linkage disequilibria may occur in natural populations (Slavov et al. 2012; Hohenlohe et al. 2012), suggesting that local heterozygosity effects could be much more widespread than previously thought. Currently, this view is gaining increasing empirical support, with local heterozygosity effects reported in many vertebrate taxa (Acevedo-Whitehouse et al. 2006; Lieutenant-Gosselin and Bernatchez 2006; Tiira et al. 2006; Charpentier et al. 2008), including several species of birds (Hansson et al. 2004; Fossøy et al. 2009; Olano-Marin et al. 2011). Taking all these into account, investigating the structure and consequences of local HFCs emerged as a new important goal of evolutionary biology, reorienting basic questions of HFC studies (Lieutenant-Gosselin and Bernatchez 2006).

The prime objective of this study was to investigate correlations between heterozygosity at seven microsatellite loci and two fitness-related traits, growth rate and nutritional condition, in great cormorant *Phalacrocorax carbo sinensis* nestlings from a medium-size inland colony in Poland. We expected that genome-wide inbreeding effects could be present in our population due to a severe bottleneck in the numbers of this tree-nesting subspecies (*P. c. sinensis*) that occurred throughout Europe in the middle of 20th century. By the early 1960s, the size of the entire northwestern European population was reduced to 800 pairs nesting in two Dutch colonies (Goostrey et al. 1998), whereas the Polish population was limited to only 150 pairs by the 1950s (Głowaciński 1992). In the early 1970s, the numbers of *P. c. sinensis* started to increase rapidly owing to a combination of relaxed human persecution, availability of new colony sites, and greatly improved food supply as the result of water eutrophication (Hagemeijer and Blair 1997), reaching over 60,000 breeding pairs in Northwest Europe by mid 1990s (Van Eerden and Gregersen 1995) and ca. 25,000 breeding pairs in Poland at the beginning of 21th century (Tomiałojć and Stawarczyk 2003). While this recent bottleneck episode could cause severe inbreeding depression across European populations, we also acknowledge that it could generate considerable linkage disequilibria, facilitating local effect of heterozygosity at neutral loci on fitness-related traits. Thus, the second aim of this study was to explore mechanisms underlying HFCs recorded within our great cormorant population, by testing whether they reflect a genome-wide general effect or a local effect of linkage disequilibrium.

## Methods

### Study Area and General Field Procedures

The study was conducted in the colony of great cormorants at Jeziorsko reservoir (51°73′N, 18°63′E), central Poland. The colony was established in 1991, when 90 breeding pairs were recorded at the site. Since then, the size of the colony gradually increased, reaching ca. 500–600 pairs during the study period (2010–2011), and ca. 800 pairs in 2013. Although the location and spatial organization of the colony changed over time, the primary breeding habitat was the riparian willow woodland dominated by the white willow *Salix alba* and the grey willow *Salix cinerea*.

For the purpose of this study we randomly selected 57 broods at the moment of hatching (29 broods in 2010 and 28 broods in 2011). As HFCs can be context-dependent and their magnitude may vary with environmental conditions and along the season (Harrison et al. 2011), our sampling period spanned over the whole main hatching period in the colony (24 and 22 days in 2010 and 2011). In the selected broods, a minor fraction of all eggs was depredated during incubation (3.6 %) and 9.1 % of eggs failed to hatch due to embryonic mortality or infertility. In all, these broods consisted of 252 eggs, 220 of which produced hatchlings sampled for molecular analysis. Blood samples for molecular analyses (ca 10 μl) were collected soon after hatching by puncturing the ulnar vein of nestlings. No brood reduction occurred before sampling. The samples were immediately suspended in 96 % ethanol and stored until laboratory analyses. When hatching of the whole brood was completed, we collected the following measurements from all the hatchlings: body mass (±1 g), wing length (±1 mm), culmen and tarsus length (both ±0.1 mm). Since great cormorants lay eggs in 1–3 day intervals (clutch size of 3–6 eggs) and start incubating from the first egg, the hatching is asynchronous and broods typically contain chicks of different sizes. To determine hatching order, all measurements collected after hatching were reduced to the first principal component (PC1) of the principal component analysis (PCA). PC1 accounted for 96.1 % of the variability in all chosen variables and all body measurements had similar contributions to PC1 (from 0.251 to 0.256). Hatching ranks were established based on size ranks assigned to each chick from PC1 values, which followed methodology used in other species of waterbirds that exhibit marked hatching asynchrony (e.g., Cash and Evans 1986), including several cormorant species (Shaw 1985; Stockland and Amundsen 1988). All chicks were tagged on the tarsus with flexible Velcro™ strips of different colours. These strips were enlarged according to the size of chicks during successive visits. At the age of 13 days all chicks were marked with individually-numbered metal rings and Velcro™ strips were removed.

### Nestling Growth Rate

Body mass of nestlings was repeatedly measured over the period of 22 days with 3- to 5-day intervals, resulting in at least five measurements per chick. It was not possible to collect measurements near fledging, as chicks older than 25 days may jump out of the nests if humans approach (Platteeuw et al. 1995; pers. observ.). To calculate nestling growth rates we fitted logistic curves of the form y = A/[1 + B × exp(−KT)] to the measurements of body mass, where y refers to the body measurement at age T, A is an asymptotic value, B is a constant of integration, and K is the growth rate constant. We used parameter K from the fitted curves as an indicator of chick growth rates. Since we stopped collecting measurements when nestlings had not yet reached asymptotic values of their body mass, the parameter A of the curve equation was constrained with the expected mean fledgling body mass of male (2379 g) or female (1946 g) great cormorant chicks (Liordos and Goutner 2008).

### Nestling Nutritional Condition

When the oldest chick in the nest was 22 ± 1 days old, we collected approximately 2 ml of blood from ulnar vein of each chick for biochemical analyses. Due to high chick mortality [mainly due to nest collapse and starvation, see Minias and Kaczmarek (2013a) for details], we collected blood samples for 157 chicks. Blood samples were placed in tubes of EDTA, kept in a cooler, and centrifuged at 3000 r.p.m. for 15 min within 8 h of collection. The plasma was separated and kept at −20 °C until analyses. Concentrations of the following plasma metabolites were analysed for all chicks with a spectrophotometer (BTS-330, BioSystems Reagents & Instruments, Barcelona, Spain) using the methods and wavelengths given in parentheses: albumin (bromocresol green, 630 nm), triglycerides (glycerol phosphate oxidase/peroxidase, 500 nm), uric acid (uricase/peroxidase, 520 nm). All parameters were analysed using commercial kits and reagents (BioSystems Reagents & Instruments, Barcelona, Spain). 10 μl of plasma was used for albumin and triglyceride measurements, while uric acid was measured in 25 μl of plasma volume, following manufacturer protocols. Repeatabilities (Lessells and Boag 1987) of measurements were high or very high (albumin: repeatability = 0.84, F_60,59_ = 20.62, *p* < 0.001; triglycerides: repeatability = 0.97, F_60,59_ = 65.10, *p* < 0.001; uric acid: repeatability = 0.97, F_60,59_ = 87.87, *p* < 0.001), based on duplicate assays for 30 randomly selected chicks.

Plasma concentrations of triglycerides and uric acid positively correlated with body mass-tarsus residuals (triglycerides: r = 0.28, *p* < 0.001; uric acid: r = 0.29, *p* < 0.001) and a similar correlation for albumin concentration was marginally significant (r = 0.15, *p* = 0.063), indicating that these parameters were likely to reliably indicate nutritional condition of cormorant nestlings. Since all three parameters were inter-correlated (all *p* < 0.001, except for the correlation between albumin and uric acid where *p* = 0.17), we reduced them to the first principal component (PC1) using PCA. The analysis was done on log-transformed data using a correlation matrix with no factor rotation. PC1 accounted for 59.1 % of variability in all univariate measurements (eigenvalue 1.77). Factor loadings were 0.73 for albumin, 0.89 for triglycerides, and 0.66 for uric acid. PC1 scores were log-transformed to improve normality, standardized to equal-unit variances (z-scores) and used as a proxy of nestling nutritional condition.

### Molecular Analyses

We performed molecular sexing and microsatellite genotyping on DNA extracted from the blood after evaporation of the alcohol (Blood Mini kit, A&A Biotechnology, Gdynia, Poland). For molecular sexing, we amplified the chromohelicase-DNA binding protein (CHD) gene with the primer pair 2550F and 2718R (Fridolfsson and Ellegren 1999), according to the protocol described by Griffiths et al. (1998). PCR products (ca. 200 bp) were separated by electrophoresis in a 2 % agarose gel stained with ethidium bromide until the differences in size were clearly visible in UV light.

For genotyping, we used seven microsatellite loci previously developed for the great cormorant (Piertney et al. 1998). Forward primers were labelled with fluorescent dye (D2, D3, D4, Sigma-Aldrich, Poland). We used multiplex procedure for five loci (PcD4, PcD6, PcT1, PcT3, PcT4) using multiplex PCR Kit (Qiagen) in 15 ul total volume, and a separate PCR for the two other loci (PcD2, PcD5) using Polimerease mix (Sigma Aldrich) in 25 μl total volume. We used a 55°C annealing temperature for both types of reactions. We genotyped the PCR products using a Beckman Coulter CEQ 8000 capillary automated sequencer at the Museum and Institute of Zoology, Polish Academy of Science (Warsaw, Poland). We scored alleles visually using the Beckman Coulter Fragment Analysis Software. 20 samples (9.1 %, n = 220) failed to amplify at one or more loci and were excluded from the data set.

### Microsatellite Markers and Heterozygosity

The mean number of alleles per locus was 27.3 (9–51 alleles) and observed heterozygosity ranged from 0.61 to 0.96 (Table 1). We could not directly test for linkage disequilibrium between the loci in our population due to high relatedness between individuals within broods and unknown relatedness between broods sampled in different years (due to possible shared parentage). However, as indicated by the genotypic analysis of great cormorants from over 20 European colonies, there was no indication of linkage disequilibrium between the loci and no consistent deviations from Hardy-Weinberg equilibrium (Goostrey et al. 1998). To estimate individual genetic diversity we calculated homozygosity by locus which weights the contribution of each locus to the homozygosity value depending on the allelic variability (Aparicio et al. 2006). As indicated by simulations, homozygosity by locus can outperform other metrics of heterozygosity, such as internal relatedness (IR, Amos et al. 2001), especially at loci with high allelic diversity (Aparicio et al. 2006). Homozygosity by locus was calculated as HL = (Σ*E*_*h*_)/(Σ*E*_*h*_ + Σ*E*_*j*_), where *E*_*h*_ and *E*_*j*_ are the expected heterozygosities of the loci that an individual bears in homozygosis (*h*) and in heterozygosis (*j*), respectively (Aparicio et al. 2006). In all the analyses we used 1 − HL, so that higher values indicate higher heterozygosity.

**Table 1:** Characterization of seven polymorphic loci used in this study with number of alleles (N_A_), allele size range, expected heterozygosity (H_e_), observed heterozygosity (H_o_)

Locus	N_A_	Size range (bp)	H_e_	H_o_
PcD2	11	168–212	0.85	0.83
PcD4	13	150–180	0.82	0.81
PcD5	17	204–262	0.82	0.82
PcD6	9	170–192	0.61	0.61
PcT1	49	287–437	0.96	0.96
PcT3	51	214–354	0.97	0.94
PcT4	41	183–295	0.95	0.91

### HFC Calculations

To analyse the effects of heterozygosity on nestling growth rate and nutritional condition we used general linear mixed models (GLMM) with nest identity included as random factor to avoid pseudoreplication (Hurlbert 1984). Since marked competitive hierarchies that arise within great cormorant broods due to hatching asynchrony are known to be a key determinant of chick growth and condition (Minias and Kaczmarek 2013b), we expected that the effects of heterozygosity may be apparent only at the between-nest level. Thus, to separate within- and between-nest effects of heterozygosity we used within-group centering, where the centered value (representing a within-nest effect) and the mean value of each nest (representing a between-nest effect) was included in the same model (van de Pol and Wright 2009). Hatching rank, hatching date and brood size were included as covariate factors in each model. Since great cormorants are sexually dimorphic in size, with males on average 10 % larger than females (Koffijberg and Van Eerden 1995), we entered nestling sex as a fixed factor in the models to account for inter-sexual variation in growth and condition. We also controlled for between-year variation in all the models. We used a stepwise procedures of backward removal to select for significant independent variables. Marginally significant effects (*p* < 0.06) were retained in the reduced models. All GLMMs were analysed with JMP Pro 10 (SAS Institute Inc., Cary, NC, USA).

### Identity Disequilibrium

General effects of heterozygosity are expected to occur only when the variance in individual inbreeding coefficients within population is large enough to cause identity disequilibrium, which is a correlation of heterozygosity or homozygosity across markers within individuals and which should reflect identity by descent (IBD) of those markers (Bierne et al. 2000; Szulkin et al. 2010; Miller and Coltman 2014). We tested for identity disequilibrium with two approaches. First, we calculated heterozygosity-heterozygosity correlation (HHC) according to the procedure described by Balloux et al. (2004). All the markers were randomly divided into two subsets of three and four loci that were separately used to calculate multi-locus heterozygosity and then the correlation coefficient between the heterozygosity of the two subsets was calculated. Significance of HHC was tested with 1000 random partitions of loci in R (R Development Core Team 2013) using the ‘h_cor’ function in the ‘Rhh’ package (Alho et al. 2010). However, this procedure was suggested to yield a complicated distribution of HHC coefficients, which are not independent from one another and, consequently, cannot provide any synthetic measure related to HFC theory (Szulkin et al. 2010). Thus, we also calculated the g_2_ statistic developed by David et al. (2007), which assesses the covariance of heterozygosity between markers standardized by their average heterozygosity. As such, g_2_ summarizes the variance in inbreeding within population, rather than individual realized IBD (Szulkin et al. 2010; Miller and Coltman 2014). g_2_ was calculated using RMES software (David et al. 2007) and its significance was tested with 1000 genotype permutations.

## Results

**Fig. 1:**
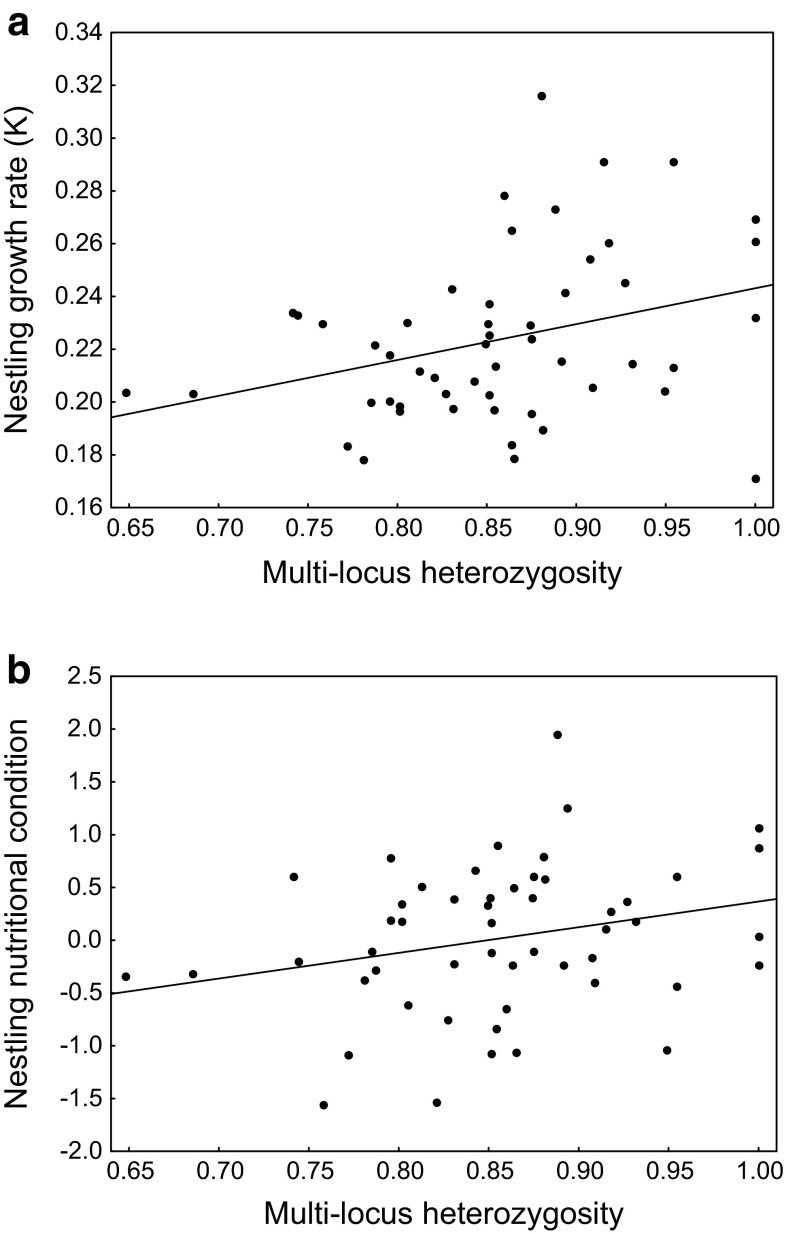
Between-nest effects (mean values for each nest) of multi-locus heterozygosity on growth rate (**a**) and nutritional condition (**b**) of great cormorant nestlings. The *lines* indicate fitted regressions

After accounting for the effects of sex, hatching rank, and year, we found a significant positive relationship between multi-locus heterozygosity and nestling growth rate at the between-nest level (Fig. 1a), while there was no significant within-nest relationship between these traits (Table 2). A similar pattern was found for nutritional condition of nestlings, with multi-locus heterozygosity showing a significant between-nest effect (Fig. 1b) and no significant within-nest effect of heterozygosity on condition (Table 3).

**Table 2:** Mixed model analyses showing within- and between-nest effects of heterozygosity on growth rates of great cormorant nestlings (n = 157)

Factor	Estimate ± SE	*t*	*p*
Full model			
Intercept	0.076 ± 0.054	1.41	0.17
Within-nest heterozygosity	0.016 ± 0.020	0.80	0.42
Between-nest heterozygosity	0.162 ± 0.053	3.05	0.004
Sex	0.006 ± 0.002	2.54	0.012
Brood size	0.006 ± 0.006	1.14	0.26
Hatching rank	−0.004 ± 0.002	−2.19	0.031
Hatching date	0.004 ± 0.004	1.11	0.27
Year	−0.010 ± 0.004	−2.70	0.010
Reduced model			
Intercept	0.135 ± 0.046	2.97	0.005
Between-nest heterozygosity	0.114 ± 0.053	2.15	0.036
Hatching rank	−0.004 ± 0.002	−1.91	0.058
Sex	0.006 ± 0.002	2.64	0.010
Year	−0.009 ± 0.004	−2.39	0.021

The results of full model and reduced model are presented. Brood identity was included as a random factor to control for non-independence among young within a brood

**Table 3:** Mixed model analyses showing within- and between-nest effects of heterozygosity on nutritional condition of great cormorant nestlings (n = 157)

Factor	Estimate ± SE	*t*	*p*
Full model			
Intercept	−1.97 ± 1.39	−1.42	0.16
Within-nest heterozygosity	0.82 ± 0.80	1.03	0.31
Between-nest heterozygosity	2.05 ± 1.36	1.51	0.14
Sex	0.00 ± 0.08	0.01	0.99
Brood size	0.08 ± 0.14	0.53	0.60
Hatching rank	−0.02 ± 0.08	−0.32	0.75
Hatching date	0.12 ± 0.10	1.23	0.23
Year	−0.17 ± 0.09	−1.76	0.09
Reduced model			
Intercept	−2.18 ± 1.09	−2.00	0.051
Between-nest heterozygosity	2.57 ± 1.28	2.01	0.049

The results of full model and reduced model are presented. Brood identity was included as a random factor to control for non-independence among young within a brood

**Fig. 2:**
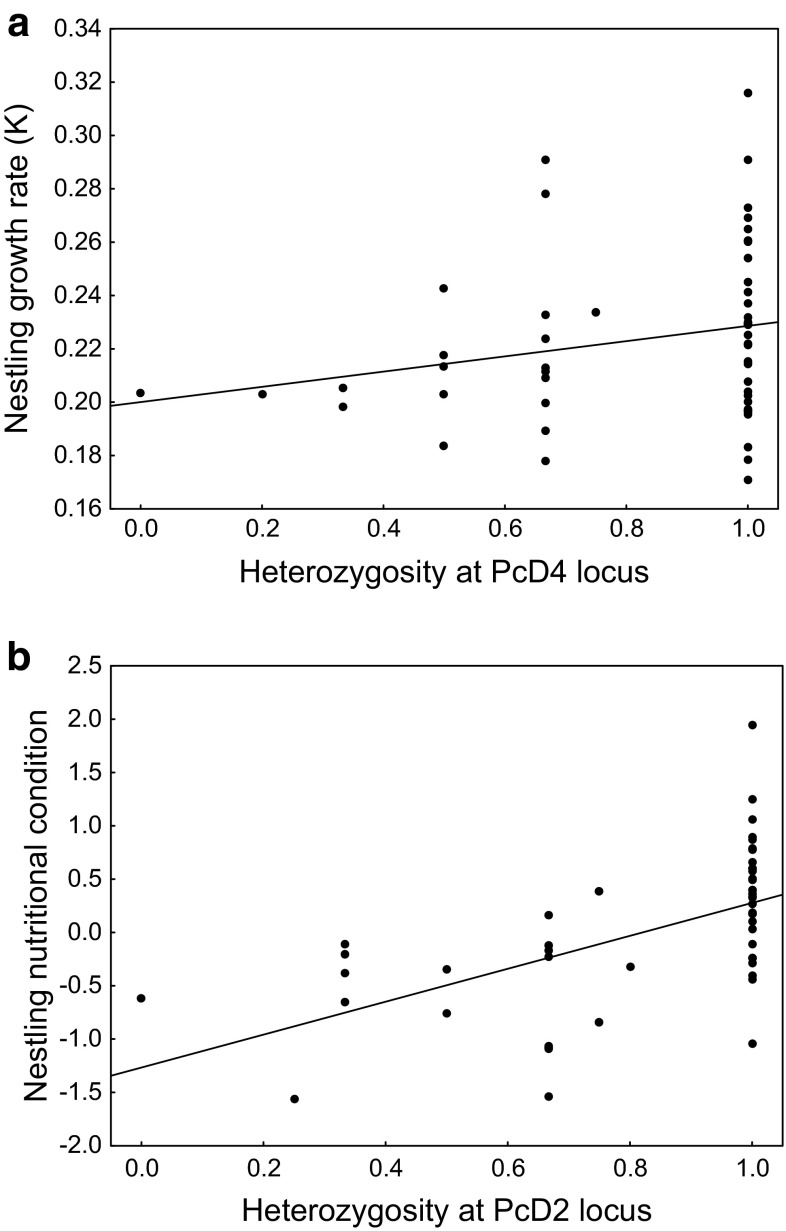
Between-nest effects (mean values for each nest) of single locus heterozygosity on growth rate (**a**) and nutritional condition (**b**) of great cormorant nestlings. The *lines* indicate fitted regressions

To test whether the HFC recorded at the between-nest level for each of fitness-related traits can be explained with the local effect hypothesis we compared a model incorporating specific heterozygosity effects separately for each locus (*m2*) with a model of multi-locus heterozygosity calculated across all loci (*m1*, following Szulkin et al. 2010). While we failed to find any significant difference in the variance explained by both models for nestling growth rate (F_6,149_ = 0.87, *p* = 0.52), there was a strong support for a local effect of heterozygosity acting on chick nutritional condition, as the model with single-locus effects explained significantly more variance than the model with heterozygosity at multiple loci (F_6,149_ = 4.49, *p* < 0.001). As indicated by the model with single-locus effects, only one of the markers (PcD2) showed significant positive association with nestling nutritional condition and, thus, appeared to contribute disproportionately toward the observed HFC (Table 4; Fig. 2b). In fact, after removing PcD2 from the estimate of multi-locus heterozygosity, the relationship between heterozygosity and nestling nutritional condition was no longer significant (F_1,104_ = 0.22, *p* = 0.64). Similarly, only one marker (PcD4) revealed a nearly significant positive association with nestling growth rate (Table 4; Fig. 2a) and multi-locus heterozygosity calculated for all other marker was not significantly related to nestling growth rate (F_1,102_ = 1.59, *p* = 0.21).

**Table 4:** Between-nest effects of single-locus heterozygosity on growth rate and nutritional condition of great cormorant nestlings

Locus	Growth rate		Nutritional condition	
	Estimate ± SE	*p*	Estimate ± SE	*p*
PcD2	0.008 ± 0.065	0.67	**1.59** ± **0.34**	<**0.001**
PcD4	**0.035** ± **0.018**	**0.057**	−0.17 ± 0.34	0.61
PcD5	0.004 ± 0.015	0.78	0.20 ± 0.29	0.49
PcD6	0.012 ± 0.013	0.35	−0.02 ± 0.24	0.93
PcT1	0.065 ± 0.038	0.11	0.50 ± 0.75	0.49
PcT3	0.011 ± 0.028	0.70	−0.01 ± 0.53	0.99
PcT4	0.021 ± 0.030	0.50	−0.14 ± 0.58	0.81

Significant and marginally significant (*p* < 0.06) values are in bold

We found no evidence for significant identity disequilibrium in our dataset. The mean within-individual HHC coefficient calculated according to Balloux et al. (2004) was negative and did not differ significantly from zero (r = −0.06, 95 % CI = −0.15 to 0.01). Consistently, the g_2_ statistic did not significantly differ from zero (g_2_ = −0.0023, *p* = 0.78), suggesting that heterozygosity measured at our set of microsatellite loci may not be representative of inbreeding.

## Discussion

We demonstrated that heterozygosity at neutral markers was positively correlated with growth and condition of great cormorant nestlings, but we also found that these relationships were primarily driven by heterozygosity at single loci. Model comparisons according to the procedure proposed by Szulkin et al. (2010) gave strong support for the local effect of heterozygosity acting on nestling nutritional condition only, but it must be kept in mind that the local effects are extremely difficult to detect with this methodology if the multi-locus heterozygosity effects are already weak (Szulkin et al. 2010). In fact, Szulkin et al. (2010) were not aware of any HFC data that passed this rigorous test and detected significant local effects. Taking all this into account, our results provide empirical evidence for the local heterozygosity effects on at least one fitness-related trait in great cormorant nestlings. Consequently, it seems likely that the loci contributing most to the observed HFCs could be at linkage disequilibrium with genes under selection, presumably with ones that play a part in regulation of growth or metabolic pathways. Such linkage disequilibria may arise in finite populations as a result of genetic drift, especially following recent bottlenecks or colonisation of new areas (Szulkin et al. 2010). In fact, we expected that the recent dramatic reduction in the size of the European *P. c. sinensis* population could have generated extensive linkage disequilibria across the genome. We found no correlation between heterozygosity at individual microsatellite loci, suggesting that our estimate of multi-locus heterozygosity did not reflect genome-wide heterozygosity, thus not supporting the ‘general effect’ hypothesis. Also, the parameter g_2_ gave no indication for inbreeding in our population. The results confirm that HFCs, at least those derived from studies examining very small number of loci, may not only be interpreted as evidence of inbreeding and that genetic associations between functional and selectively neutral markers could be much more common in natural populations than previously thought.

In this study, local effects of heterozygosity were found only at the between-brood level with no individual effects apparent within broods. This pattern was expected in the great cormorant, because the pronounced hatching asynchrony of nestlings (hatching period lasting up to 5 days in large broods) results in marked competitive asymmetries within broods. Great cormorant nestlings that hatch later in the hatching sequence have lower access to food delivered by parents, which leads to significant reductions in body mass and slower growth rates in comparison to older siblings. In our population, a large proportion of variation in chick body mass (81 %) and concentration of plasma metabolites (68–76 %) was attributed to within-brood differences (Minias and Kaczmarek 2013b). Consequently, it seems that within-brood effects of heterozygosity could be masked by very strong effects of competitive hierarchies among young. Taking this into account, we suggest that within-brood effects of heterozygosity are much more likely to be found in species where offspring hatch synchronously. Consistent with this prediction, there is empirical evidence for within-brood effects of heterozygosity on such fitness-related traits as survival (Hansson et al. 2001; Townsend and Jamieson 2013) or cell-mediated immunity (Fossøy et al. 2009; Voegeli et al. 2013) in synchronously or nearly-synchronously hatching passerines. Although both general and local effects were suggested to underlie these relationships, it has been recently pointed out that within-brood HFCs should be interpreted with care, as full-siblings can vary in the proportion of the genome that is identical by descent due to chance events during Mendelian segregation, and such variation may be sufficient to cause HFCs among full-sibs (Forstmeier et al. 2012). Interpretation of within-brood HFCs may also be hampered by the effects of mixed paternity (Townsend and Jamieson 2013). While there is a molecular evidence for extra-pair paternity (EPP) in the Great Cormorant, its frequency was reported to vary substantially between colonies (Piertney et al. 2003) and no information on the level of EPP was available for our studied population.

We cannot exclude the possibility that there was also an indirect effect of parental genetic quality contributing to the HFCs found in this cormorant population. Genetic diversity is theoretically expected to be correlated between parents and offspring, since at most allelic frequencies heterozygous parents produce higher proportions of heterozygous progeny than homozygous parents (Mitton et al. 1993). Positive associations between parental and offspring heterozygosity were empirically demonstrated for birds (García-Navas et al. 2009) and other vertebrate species (Cothran et al. 1983; Hoffman et al. 2007). Thus, fitness benefits associated with genetic diversity can be partly heritable, but the effect of parental heterozygosity on nestling development may also have a non-genetic component. Heterozygous parents may have increased competitive abilities (Höglund et al. 2002; Minias et al. 2015), which, in turn, can increase their foraging efficiency and elevate the amount of food delivered to offspring, thus having a positive impact on their growth and condition. Alternatively, adults paired with genetically dissimilar mates may increase their parental investment in offspring, which can amplify complementary gene effects and drive enhanced nestling growth rates or condition (Potvin and MacDougall-Shackleton 2009; Arct et al. 2010). As a consequence, studies with an appropriate experimental design would be necessary to explicitly separate non-genetic parental effect from genetic effects of heterozygosity on fitness-related traits of their progeny.
